# Informatics Interventions for Maternal Morbidity: Scoping Review

**DOI:** 10.2196/64826

**Published:** 2025-03-25

**Authors:** Jill Inderstrodt, Julia C Stumpff, Rebecca C Smollen, Shreya Sridhar, Sarah A El-Azab, Opeyemi Ojo, Brendan Bowns, David A Haggstrom

**Affiliations:** 1 Department of Health Policy and Management Richard M. Fairbanks School of Public Health Indiana University Indianapolis, IN United States; 2 Center for Biomedical Informatics Regenstrief Institute Indianapolis, IN United States; 3 Center for Health Information and Communication Richard L. Roudebush VA Medical Center Indianapolis, IN United States; 4 Ruth Lilly Medical Library Indiana University School of Medicine Indianapolis, IN United States; 5 Department of Public Health College of Health and Human Sciences Purdue University West Lafayette, IN United States; 6 School of Pharmacy University of Pittsburgh Pittsburgh, PA United States; 7 Department of Health Policy and Management University of Michigan Ann Arbor, MI United States; 8 Department of Epidemiology Richard M. Fairbanks School of Public Health Indiana University Indianapolis, IN United States; 9 Department of Prevention Corktown Health Detroit, MI United States; 10 Center for Health Services Research Regenstrief Institute Indianapolis, IN United States; 11 Division of General Internal Medicine and Geriatrics Department of Medicine Indiana University School of Medicine Indianapolis, IN United States

**Keywords:** scoping review, maternal morbidity, medical informatics, clinical informatics, mother, pregnant, perinatal, GDM, preeclampsia, maternity, gestational diabetes mellitus

## Abstract

**Background:**

Women have been entering pregnancy less healthy than previous generations, placing them at increased risk for pregnancy complications. One approach to ensuring effective monitoring and treatment of at-risk women is designing technology-based interventions that prevent maternal morbidities and treat perinatal conditions.

**Objective:**

This scoping review evaluates what informatics interventions have been designed and tested to prevent and treat maternal morbidity.

**Methods:**

MEDLINE, Embase, and Cochrane Library were searched to identify relevant studies. The inclusion criteria were studies that tested a medical or clinical informatics intervention; enrolled adult women; and addressed preeclampsia, gestational diabetes mellitus (GDM), preterm birth, Centers for Disease Control and Prevention–defined severe maternal morbidity, or perinatal mental health conditions. Demographic, population, and intervention data were extracted to characterize the technologies, conditions, and populations addressed.

**Results:**

A total of 80 studies were identified that met the inclusion criteria. Many of the studies tested for multiple conditions. Of these, 73% (60/82) of the technologies were tested for either GDM or perinatal mental health conditions, and 15% (12/82) were tested for preeclampsia. For technologies, 32% (28/87) of the technologies tested were smartphone or tablet applications, 26% (23/87) were telehealth interventions, and 14% (12/87) were remote monitoring technologies. Of the many outcomes measured by the studies, almost half (69/140, 49%) were patient physical or mental health outcomes.

**Conclusions:**

Per this scoping review, most informatics interventions address three conditions: GDM, preeclampsia, and mental health. There may be opportunities to treat other potentially lethal conditions like postpartum hemorrhage using proven technologies such as mobile apps. Ample gaps in the literature exist concerning the use of informatics technologies aimed at maternal morbidity. There may be opportunities to use informatics for lesser-targeted conditions and populations.

## Introduction

Women have been entering pregnancy less healthy than previous generations [1]. Maternal morbidities such as preeclampsia and gestational diabetes, and severe maternal morbidities such as postpartum hemorrhage all have implications for the long-term health of mothers well beyond the postpartum period. Preeclampsia is associated with cardiovascular disease for decades following pregnancy, including chronic hypertension, stroke, and age-adjusted overall mortality [2]. Gestational diabetes mellitus (GDM), a condition that in many cases resolves itself after delivery, can still affect mothers beyond the puerperium [3]. Severe maternal morbidity (SMM) encompasses myriad conditions, including acute myocardial infarction, eclampsia, and hemorrhage. Those who have experienced SMM are more likely to die at any point after delivery and into the decades beyond the postpartum period [4]. Preterm birth (PTB; delivering before 37 weeks gestation) is associated with long-term cardiovascular complications in the mother, including ischemic heart disease, stroke, and atherosclerosis [5].

Informatics interventions offer tools that can help to prevent perinatal health conditions that have long-term health consequences for mothers, monitor these conditions in the perinatal period so that mother and baby remain healthy, and follow mothers post partum to ensure that they continue to receive the health monitoring they need. Technology-based tools that can be used for these purposes include mobile apps, wearable technology, physician decision support, and telehealth, among others. As artificial intelligence (AI) technology becomes more common in health care, a range of interventions can be used to predict, diagnose, and treat maternal morbidities that lead to maternal complications, with the hope of reducing maternal morbidity and mortality [6]. While some patients are hesitant for their physicians to use AI to diagnose or treat medical problems, physicians have demonstrated growing acceptance and belief that AI will improve interactions with their patients [7]. As the landscape of health informatics shifts substantially with the advent of new technologies, it is important to assess what technologies have already been tested to prevent, diagnose, and treat common maternal morbidities so that gaps can be identified and addressed in future research [8].

In this scoping review, we seek to identify what informatics interventions have been designed and tested that address maternal morbidity. In addition, we aim to assess the extent, range, and nature of informatics interventions that have been tested to prevent, diagnose, and treat maternal morbidities known to have long-term health consequences for mothers. The review summarizes discrete populations addressed in the literature, types of informatics interventions tested in the literature, conditions addressed by these interventions, and outcomes measured.

## Methods

### Search Strategy

Our scoping review methodology was guided by Arksey and O’Malley’s [9] framework, stages 1-5, and Joanna Briggs’s *Manual for Scoping Reviews*. We asked what informatics interventions have been designed and tested for SMM. Using the PCC (Population, Concept, and Context) framework outlined in the *Manual for Scoping Reviews*, the population was adult women with SMM as defined by the Centers for Disease Control and Prevention (CDC), the concept was informatics interventions, and the context was prospective studies anywhere in the world. We developed a review protocol to guide the process. The literature searches were led by an expert information specialist (JCS) in consultation with the research team. Four electronic databases were searched from inception until June 6, 2022: MEDLINE and Embase (searched simultaneously on Ovid), Cochrane Library (Wiley), and Engineering Village (Elsevier). One database was searched from inception until June 7, 2022: IEEE Xplore (IEEE). The MEDLINE (Ovid) search was peer-reviewed by another expert librarian using the PRESS (Peer Review of Electronic Search Strategies) checklist and modified as required [10]. The search strategy was limited to the English language and encompassed all years of publication. Embase and MEDLINE citations were deduplicated in Ovid before exporting to Covidence online review software. The full search strategies for each database are publicly available in searchRxiv [11]. Lastly, review results are reported using PRISMA-ScR (Preferred Reporting Items for Systematic Reviews and Meta-Analyses Extension for Scoping Reviews) [12]. This checklist can be found in Multimedia Appendix 1. Our protocol was registered retrospectively in Open Science Framework Registries [13].

### Study Selection

The inclusion and exclusion criteria are described in Textbox 1. Studies were included that prospectively tested a medical informatics intervention on either a group of women or physicians who treat women. Study methodologies could include both quantitative and qualitative approaches. Only studies analyzing primary data to describe a health-related outcome were included. Studies were included that tested medical or clinical informatics interventions on human subjects to prevent, monitor, or treat maternal conditions that have long-term health consequences for mothers within and beyond the perinatal period. Conditions included preeclampsia or hypertensive disorders of pregnancy, GDM, PTB, new mental health diagnoses, and SMM as defined by the US CDC [14].

**Criteria for inclusion and exclusion.**

**Inclusion criteria**

- Test a medical informatics intervention prospectively
- Medical or clinical informatics interventions
- Adult women or physicians who treat women
- Preeclampsia, gestational diabetes mellitus, preterm birth, perinatal mental health diagnoses, severe maternal morbidity as defined by the Centers for Disease Control and Prevention (21 indicators)
- Physical health, patient-centered outcomes, mental health, health behavior, health knowledge or attitudes, health care use, quality of care outcomes

**Exclusion criteria**

- Systematic reviews, scoping reviews, literature reviews, opinion pieces, commentaries, proposals, reports, conference papers
- Pilot/feasibility studies
- Bioinformatics studies, including scans, ultrasounds, biomarkers, and predictive algorithms
- Adolescents
- Preexisting conditions: type 1 or 2 diabetes, chronic hypertension, preexisting mental health conditions
- Feasibility, acceptability, user experience

Systematic reviews, scoping reviews, and literature reviews were excluded together with opinion pieces, commentaries, proposals, reports, or gray literature. Studies were also excluded if they focused primarily on bioinformatics, effectively eliminating imaging (eg, ultrasounds), biomarkers, and predictive algorithm development.

Article references were entered into review software for screening and data extraction. The first and senior authors conducted an initial title, abstract, and keyword screening. If any author recommended inclusion, then the article underwent full-text screening. All authors except for the information specialist (JCS) then conducted a full-text screening, with two team members reviewing all articles. Studies with two votes to include were moved forward for extraction; those with two votes to exclude were excluded from the study. During the full-text screening, Cohen κ was used in the initial training to gauge agreement until reviewers reached a κ of 0.80. After training, disagreements were resolved by the first author.

### Data Extraction

A subset of four authors extracted data using Covidence review software. Reviewers were given an initial set of 10 articles and met with the first author for consensus before reviewing the remaining articles. All studies were reviewed for extraction by the first author and one additional author. Most data extraction categories were developed a priori, and selection options were developed by the first and senior authors after reviewing all articles for trends. The following data categories were added after the protocol had been developed: aim of the study, years of data collection, population inclusion and exclusion criteria, and total number of participants. These categories were added to the data extraction form iteratively to provide more context to the reviewed studies.

Demographic data collected for each article by reviewers included title of article, year published, lead author surname, and country in which the study was conducted. The following characteristics of included studies were extracted: informatics technology tested, health problem, aim of study, study design, years of data collection, population description, study participation inclusion criteria, study participation exclusion criteria, total number of participants, and health outcome. Reviewers were given the option of selecting multiple answers for each study characteristic. An open-ended “other” option was available for the following study characteristics: informatics technology, health problem, study design, and outcome. Following extraction, the first and senior authors also extracted data on whether a significantly positive result was found in the studies, as well as racial diversity in participant samples for those studies conducted in the United States.

Data were synthesized by tallying the options from each category (study design, region, technology, health outcome, study population, study outcome, and results) and calculating the corresponding frequencies for each option.

## Results

### Overview

**Figure 1:**
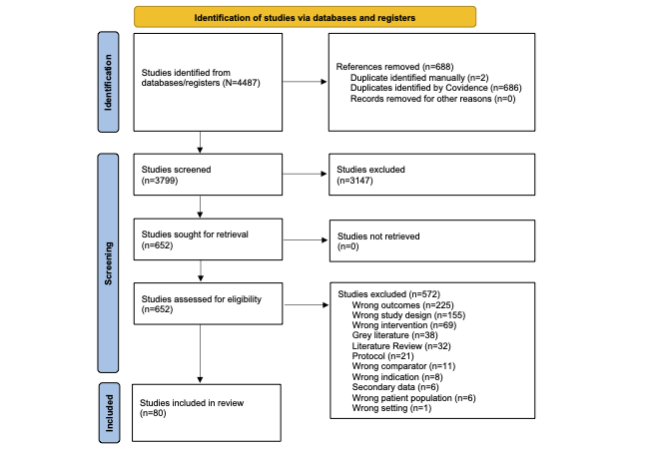
PRISMA (Preferred Reporting Items for Systematic Reviews and Meta-Analyses) diagram.

The PRISMA flow diagram for this scoping review can be found in Figure 1. A total of 3799 records were identified for title and abstract screening; 652 records underwent full-text screening for eligibility. Of these, 572 were excluded. Common reasons for exclusion were study design (eg, retrospective, systematic or other review, narrative), type of study outcome (user experience), or the wrong intervention (eg, machine learning algorithms or predictive models not tested with patients or providers). A total of 80 studies underwent full data extraction. A list of the studies and their characteristics can be found in Table 1.

Frequencies for synthesized data can be found in Table 2.

**Table 1:** Studies selected for extraction.

Author (year)	Country	Technology	Condition	Study design	Population	Sample size, n	Outcome
Abbaspoor et al [15] 2020	Iran	SMS text message	GDM^a^	RCT^b^	Women	100	Medical Health behavior
Abejirinde et al [16] 2019	Ghana	Smartphone/tablet application DSSc	HDP^d^, GDM	Nonrandomized experimental	Women	940	Quality of care
Al-Ofi et al [17] 2019	Saudi Arabia	Remote monitoring Telehealth	GDM	RCT	Women	57	Medical Health behavior
Arias et al [18] 2022	United States	Telehealth	HDP	Cohort	Women	1579	Health behavior Health care use Quality of care
Bartholomew et al [19] 2015	United States	Remote monitoring Smartphone/tablet application Web program	GDM	RCT	Women	74	Number of readings
Baumel et al [20] 2018	United States	Smartphone/tablet application Web program	Mental health condition	Nonrandomized experimental	Women	19	Mental health
Bellad et al [21] 2020	India	Web application	HDP	RCT	Providers	14,783	Medical
Borgen et al [22] 2019	Norway	Smartphone/tablet application	GDM	RCT	Women	158	Medical Health behavior
Carlisle et al [23] 2022	United Kingdom	DSS	Mental health condition, PTB^e^	RCT	Women	300	Mental health
Carroll et al [24] 2013	United States	DSS	Mental health condition	RCT	Providers	48	Quality of care
Chan et al [25] 2019	China	Smartphone/tablet application	Mental health condition	RCT	Women	660	Mental health
Chappell et al [26] 2022	United Kingdom	Remote monitoring	HDP	RCT	Women	454	Medical
Cheung et al [27] 2019	Australia	SMS text messaging Wearable	GDM	RCT	Women	60	Medical Number of readings Health behavior
Dennis et al [28] 2009	Canada	Telehealth	Mental health condition	RCT	Women	846	Medical Mental health
Dennis et al [29] 2020	United States	Telehealth	Mental health condition	RCT	Women	249	Medical Mental health Health care use
Felder et al [30] 2017	United States	Web program	Mental health condition	Nonrandomized experimental	Women	27	Medical Mental health
Ferrara et al [31] 2012	United States	Telehealth	GDM	RCT	Women	1000	Medical Number of readings
Ferrara et al [32] 2016	United States	Telehealth	GDM	RCT	Women	523	Medical Health behavior
Forsell et al [33] 2017	Sweden	Telehealth Web program	Mental health condition	RCT	Women	72	Patient-centered outcomes Mental health
Garnweidner-Holme et al [34] 2020	Norway	Remote monitoring Smartphone/tablet application	GDM	RCT	Women	238	Health behavior
Ghaderi et al [35] 2019	Iran	Smartphone/tablet application	GDM	RCT	Women	134	Health knowledge/attitudes
Goetz et al [36] 2020	Taiwan	Smartphone/tablet application	Mental health condition	Cohort	Women	30	Mental health
Gong et al [37] 2021	China	SMS text messaging	Mental health condition	RCT	Women	291	Medical Mental health
Guille et al [38] 2021	United States	SMS text messaging Telehealth	Mental health condition	Nonrandomized experimental	Women	2988	Quality of care Health behavior Health care use
Guo et al [39] 2019	China	Smartphone/tablet application	GDM	RCT	Women	172	Medical Number of readings Health behavior Health care use
Hantsoo et al [40] 2018	United States	Smartphone/tablet application	Mental health condition	RCT	Women	61	Health behavior Health care use Quality of care
Hedderson et al [41] 2018	United States	DSS	GDM	RCT	Women	2014	Medical
Heller et al [42] 2020	Netherlands	Web program	Mental health condition	RCT	Women	159	Mental health
Homko et al [43] 2007	United States	Web program	GDM	RCT	Women	57	Medical Number of readings Health knowledge/attitudes
Homko et al [44] 2012	United States	Telehealth	GDM	RCT	Women	253	Medical Number of readings
Hoppe et al [45] 2020	United States	Telehealth	HDP	Nonrandomized experimental	Women	428	Medical Number of readings Health care use
Huang et al [46] 2021	China	Smartphone/tablet application	GDM	RCT	Women	309	Medical
Jannati et al [47] 2020	Iran	Smartphone/tablet application	Mental health condition	RCT	Women	75	Mental health
Klokkenga et al [48] 2019	Ghana	Smartphone/tablet application	Hemorrhage	RCT	Women	146	Medical
Krishnamurti et al [49] 2021	United States	Smartphone/tablet application	HDP	Cohort	Women	2567	Health behavior Quality of care
Lanssens et al [50] 2017	Belgium	Remote monitoring	HDP	Retrospective	Women	166	Medical Health care use Quality of care
Lanssens et al [51] 2018	Belgium	Remote monitoring	HDP	Retrospective	Women	320	Medical Health care use Quality of care
Latendresse et al [52] 2021	United States	Telehealth	Mental health condition	Nonrandomized experimental	Women	47	Mental health
Lewey et al [53] 2022	United States	SMS text messaging Web program Wearable	HDP	RCT	Women	127	Medical Health behavior
Li et al [54] 2021	China	Telehealth	GDM	RCT	Women	287	Medical Health behavior
Lim et al [55] 2021	Singapore	Smartphone/tablet application	GDM	RCT	Women	200	Medical Mental health
Mackillop et al [56] 2018	United Kingdom	Telehealth SMS text messaging	GDM	RCT	Women	203	Medical Number of readings Patient-centered outcomes
Milgrom et al [57] 2016	Australia	Web program	Mental health condition	RCT	Women	43	Mental health
Milgrom et al [58] 2021	Australia	Telehealth	Mental health condition	RCT	Women	116	Mental health
Miremberg et al [59] 2018	Israel	Smartphone/tablet application	GDM	RCT	Women	120	Medical Number of readings Patient-centered outcomes Health behavior
Missler et al [60] 2020	Netherlands	Telehealth	Mental health condition	RCT	Women	130	Mental health Parenting measures
Ngai et al [61] 2015	China	Telehealth	Mental health condition	RCT	Women	397	Mental health
Nieminen et al [62] 2016	Sweden	Web program	Mental health condition	RCT	Women	43	Patient-centered outcomes Mental health Health behavior
Niksalehi et al [63] 2018	Iran	SMS text messaging	Mental health condition	Nonrandomized experimental	Women	54	Mental health
Nishimwe et al [64] 2021	Rwanda	Smartphone/tablet application	Hemorrhage	Nonrandomized experimental	Providers	54	Health knowledge/attitudes Quality of care
Parsa et al [65] 2019	Iran	Smartphone/tablet application	HDP	Nonrandomized experimental	Women	110	Health knowledge/attitudes
Perry et al [66] 2018	United Kingdom	Remote monitoring Smartphone/tablet application	HDP	Case control	Women	108	Medical Number of readings Health care use
Posmontier et al [67] 2016	United States	Telehealth	Mental health condition	Cohort	Women	61	Patient-centered outcomes Mental health Quality of care
Potzel et al [68] 2022	Germany	Smartphone/tablet application	GDM	RCT	Women	203	Medical Number of readings Health behavior
Rahman et al [69] 2017	Bangladesh	Web program	Hemorrhage	RCT	Women	310	Quality of care
Rasekaba et al [70] 2018	Australia	Telehealth	GDM	RCT	Women	95	Medical Health care use
Sawyer et al [71] 2019	Australia	Smartphone/tablet application	Mental health condition	RCT	Women	133	Mental health
Shamshiri Milani et al [72] 2015	Iran	Telehealth	Mental health condition	RCT	Women	126	Mental health
Silva-Jose et al [73] 2021	Spain	Telehealth Web program	HDP	RCT	Women	206	Medical Health behavior
Simhi et al [74] 2021	Israel	Telehealth	Mental health condition	RCT	Women	77	Mental health Health behavior
Skar et al [75] 2018	Norway	Smartphone/tablet application	GDM	Qualitative	Women	17	Mental health Health knowledge/attitudes
St Pierre et al [76] 2017	Germany	Anesthesia machine cognitive aid	Myocardial infarction	RCT	Women	83	Quality of care
Sun et al [77] 2021	China	Smartphone/tablet application	Mental health condition	RCT	Women	186	Mental health
Sung et al [78] 2019	Korea	Remote monitoring Smartphone/tablet application	GDM	RCT	Women	21	Medical
Sung et al [79] 2021	Korea	Telehealth	GDM	Cohort	Women	176	Medical Health care use
Tian et al [80] 2020	China	Smartphone/tablet application	GDM	RCT	Women	169	Health behavior
Tian et al [81] 2021	China	Telehealth	GDM	RCT	Women	309	Medical
Triebwasser et al [82] 2020	United States	SMS text messaging Web-based program	HDP	RCT	Women	333	Number of readings
Tucker et al [83] 2022	United Kingdom	Remote monitoring Smartphone/tablet application	HDP	RCT	Women	2346	Medical Mental health Quality of care
Ugarriza and Schmidt [84] 2006	United States	Telehealth	Mental health condition	RCT	Women	30	Mental health
Vandenberk et al [85] 2019	Belgium	Remote monitoring	HDP	RCT	Women	108	Number of readings Mental health
van den Heuvel et al [86] 2020	Netherlands	Remote monitoring Smartphone/tablet application	HDP	Case control	Women	133	Medical Health care use
van Montfort et al [87] 2020	Netherlands	Web program	HDP	Cohort	Women	850	Quality of care
Van Ryswyk et al [88] 2015	New Zealand	SMS text messaging	GDM	RCT	Women	268	Medical Health behavior
Vigod et al [89] 2021	Canada	Web program	Mental health condition	RCT	Women	98	Mental health
Wozney et al [90] 2017	Canada	Telehealth	Mental health condition	RCT	Women	62	Mental health
Yang et al [91] 2018	China	Smartphone/tablet application	GDM	RCT	Women	107	Medical
Yew et al [92] 2021	Singapore	Smartphone/tablet application	GDM	RCT	Women	340	Medical
Zera et al [93] 2015	United States	DSS	GDM	RCT	Women	847	Quality of care
Zhuo et al [94] 2022	China	Smartphone/tablet application	GDM	RCT	Women	124	Medical Health behavior

^a^GDM: gestational diabetes mellitus.

^b^RCT: randomized controlled trial.

^c^DSS: decision support system.

^d^HDP: hypertensive disorders of pregnancy.

^e^PTB: preterm birth.

**Table 2:** Outcomes measured.

		Frequency, n (%)
**Study design (n=80)**		
	Randomized controlled trial	60 (75)
	Nonrandomized experimental	9 (11)
	Cohort	6 (8)
	Case control	2 (3)
	Retrospective	2 (3)
	Qualitative	1 (1)
**Region (n=80)**		
	North America	24 (30)
	East Asia and Pacific	22 (28)
	Europe and Central Asia	20 (25)
	Middle East and North Africa	9 (11)
	Sub-Saharan Africa	3 (4)
	South Asia	2 (3)
	Latin America and Caribbean	0 (0)
**Technology (n=87)**		
	Application	28 (32)
	Telehealth/telemedicine	23 (26)
	Remote monitoring	12 (13)
	Web-based program	10 (11)
	SMS text messaging	8 (9)
	EHR^a^ DSS^b^	3 (3)
	Wearable	2 (2)
	Other	1 (1)
**Perinatal health outcome (n=82)**		
	New mental health diagnosis	30 (37)
	GDM^c^	30 (37)
	Preeclampsia/HDP^d^	12 (15)
	Hypertension	5 (6)
	Hemorrhage	3 (4)
	Preterm birth	1 (1)
	Myocardial infarction	1 (1)
**Study population (n=80)**		
	Women	77 (96)
	Providers	3 (4)
**Outcomes (n=140)**		
	Medical outcomes	39 (27.9)
	Mental health outcomes	30 (21.4)
	Health behavior	21 (15.0)
	Quality of care	19 (13.6)
	Number of readings	13 (9.3)
	Health care use	12 (8.6)
	Health knowledge/attitudes	5 (3.6)
	Other	1 (0.7)
**Results (n=80)**		
	Significant for at least one measure	63 (79)
	No significance	17 (21)

^a^EHR: electronic health record.

^b^DSS: decision support system.

^c^GDM: gestational diabetes mellitus.

^d^HDP: hypertensive disorders of pregnancy.

Included studies were mostly RCTs (n=60). Other study designs included nonrandomized experimental (n=9), cohort (n=6), case control (n=2), retrospective (n=2), and qualitative (n=1). Included studies represented a range of countries and regions. Most of the studies were conducted in North America (n=24), East Asia and the Pacific (n=22), and Western Europe (n=20). Fewer studies were authored from the Middle East and North Africa (n=9), sub-Saharan Africa (n=3), and South Asia (n=2). No studies focused on Latin America or the Caribbean.

### Informatics Technologies

The specific informatics technologies tested by the study were described. Descriptive categories included telehealth or telemedicine (remote consultation via telephone or video chat that may replace medical consultation), remote monitoring (patient-performed measurements of vital signs and blood glucose), electronic health record decision support system (tools within the electronic health record that assist providers with clinical decisions), smartphone or tablet applications, SMS text messages (between providers and patients or between patients), web-based programs (patient portals and other programs administered through a web browser, not through a smartphone or tablet application), and other (with the technology charted manually). Studies tested a range of informatics technologies. The most frequently tested technologies included smartphone/tablet applications (n=28) and telehealth/telemedicine (n=23). Technologies tested less frequently included remote monitoring (n=15), web-based programs (n=10), and SMS text messaging (n=8). Technologies assessed in very few studies included electronic health record decision support (n=3), wearable technologies (n=2), and other (cognitive aid on an anesthesia machine; n=1).

### Health Problems

Articles were categorized by the health problems treated by the informatics technology; some tested more than one. These included preeclampsia or hypertensive disorders of pregnancy, hypertension, GDM, PTB, hemorrhage, and new (perinatal) mental health diagnoses. Although our protocol searched for “severe maternal morbidity as defined by the CDC” and included a list of 21 conditions [13], after reviewing the articles, it was clear that the only condition from the CDC list that was frequently tested by informatics technology was postpartum hemorrhage. Thus, any additional conditions were added manually in the “other” option. Studies primarily used informatics technologies to address new mental health conditions (n=30) and GDM (n=30). Other health problems addressed by the studies include preeclampsia or hypertensive disorders of pregnancy (n=12), hypertension (n=5), and hemorrhage (n=3). One study each addressed preterm birth and myocardial infarction.

### Population Type

Population type was categorized as either: women or medical providers who treat women. Included studies overwhelmingly tested informatics technologies on women, with only 3 studies testing informatics technologies on providers. US studies were also coded for sample demographics.

### Outcomes

Finally, outcomes were categorized to gain an understanding of the measures upon which the studies focused; many tested more than one measure. These included medical outcomes (eg, weight loss, blood pressure, blood glucose), number of remote measures (eg, patient-collected and -reported blood pressure or blood glucose measurements), mental health outcomes (eg, Edinburgh Postnatal Depression Scale score, subjective well-being measures), health behaviors (eg, dietary habits, exercise frequency), health knowledge or attitudes (eg, patient’s understanding of health condition, patient-reported attitude toward care), health care use (eg, number of emergency room visits, number of prenatal care visits), quality of care (eg, Hospital Consumer Assessment of Healthcare Providers and Systems [HCAHPS] scores, safety of care, effectiveness of care, efficiency of care), or other. Studies measured myriad outcomes, and many studies measured more than one outcome. Many assessed medical outcomes (n=39), mental health outcomes (n=30), and patient health behaviors (n=21). Fewer measured quality of care (n=19), number of remote readings (n=13), and health care use (n=12). Very few measured health knowledge and attitudes (n=5) or other outcomes (n=1).

### Effect

Studies were coded as to whether a significant positive effect was exhibited for at least one health outcome measure (yes/no). Most (n=63) studies found that the informatics technology yielded at least one positive effect for a health-related (ie, not user experience) outcome. Some (n=17) studies showed no significant positive effect of the informatics technology tested.

## Discussion

### Principal Findings

This scoping review was performed to characterize what informatics interventions have been tested to address the maternal morbidities that result in long-term negative health outcomes for mothers. Our results show that most of the tested informatics technologies were tested in North America, East Asia, and Western Europe. They overwhelmingly focused on patient populations diagnosed with GDM, mental health conditions, and preeclampsia. Most were patient focused and most aimed to prevent, diagnose, or treat physical or mental health outcomes.

Women have been entering pregnancy less healthy than previous generations. As the world population grows increasingly obese, high BMI presents critical risks for birthing people, with obesity linked to such perinatal complications as gestational diabetes [95], preeclampsia [96], and postpartum hemorrhage [97]. Women also currently enter pregnancy in poorer cardiovascular health than previous generations, with prepregnancy hypertension especially pronounced in rural populations of childbearing age [98]. This scoping review surveys the landscape of informatics interventions tested to treat these increasingly common conditions.

Most of the technologies were tested in North America, East Asia or the Pacific, and Western Europe. Despite some evidence of resistance to new technologies [99], studies generally show perceptions of mobile health to be positive in sub-Saharan Africa, especially toward maternal health applications [100]. There may be opportunities to test technologies developed in North America, Asia, and Europe in underserved parts of the world. In addition to improving maternal health in these underserved areas, testing in diverse types of populations may yield compelling findings that could be translated into US, Asian, and European contexts through a process of reciprocal innovation [101].

Our results show that most of the tested informatics technologies focused on patient populations diagnosed with GDM; mental health conditions; and, to a lesser extent, preeclampsia. Far fewer focused on preventing or treating postpartum hemorrhage. Although prevalences of GDM and mental health conditions are high, preeclampsia and hemorrhage are leading causes of maternal mortality and pregnancy-associated deaths in both the United States and abroad [101]. Postpartum hemorrhage, in particular, is the leading cause of maternal death worldwide, causing 94% of maternal deaths [102,103].

There are at least two possible reasons for this imbalance in testing of interventions. First, diabetes mellitus and mental health conditions are also common in the general population, unlike preeclampsia and hemorrhage. Thus, preexisting interventions may be more easily transferrable to pregnant populations. Second, GDM and postpartum depression can be diagnosed through routine screening, with GDM diagnosed via prenatal glucose testing and postpartum depression diagnosed using the Edinburgh Postnatal Depression Scale. Indeed, 9.3% of interventions measured the extent/accuracy with which patients review their own biodata, including blood glucose levels. Screening for GDM could be seen as a parsimonious method to improve women’s health. On the other hand, postpartum hemorrhage is less predictable, typically being diagnosed through an urgent/emergent presentation. Thus, the ease of routine screening practices may lend certain diagnoses to being more amenable to the implementation of informatics interventions.

Most of the interventions in this review aimed to prevent, diagnose, or treat physical or mental health outcomes, and almost all were patient focused. Very few interventions (n=3, 4%) were tested on providers who treat women. Of those studies, two of the interventions focused on midwives and one used a decision support system to assist physicians in screening for maternal depression. Most interventions in this review were tested on women and involved active participation from patients (eg, taking blood glucose readings, logging symptoms in an app, participating in an online support group). In other words, most of the interventions entrusted patients with aspects of their own health.

This focus on outcomes is logical given the maternal mortality crisis both within and outside of the United States. Mental health conditions are the leading cause of pregnancy-related deaths in the United States according to state Maternal Mortality Review Committees [104] and of non-Hispanic White and Hispanic women when stratified by ethnicity. Cardiac conditions are the leading cause of pregnancy-related death for Black/African American women in the United States, but the focus on interventions aimed at patients themselves minimizes the role provider knowledge and practice within a health system plays in the crisis. Research shows that lack of standardized emergency obstetric care, maternity care coordination [105], and systemic racism [106] can play a role in the safe and effective delivery of care. The results of this scoping review show a large gap in the literature related to using informatics to improve the quality of care for maternal morbidities. Interventions such as telehealth have been shown to improve obstetric quality, including perinatal smoking cessation and breastfeeding [107]. It is possible that expanding such technologies and examining the way they are used by physicians can help improve the quality of care and ultimately save the lives of women.

The need for informatics interventions does not end with birth. Prior research suggests that many physicians, including obstetricians, are unaware of how maternal morbidity can influence maternal health in the decades following pregnancy [108]. Because of this lack of awareness, patients often do not know to monitor their own health in the long term, and they may not receive referrals for proper follow-up services. The paucity of provider-focused technologies identified in this review may also present opportunities for the development of informatics-based interventions targeting physicians and affiliated providers—especially in the treatment and prevention of postpartum preeclampsia and hemorrhage—that could use technologies like decision support to improve maternal health.

### Limitations

Our study has several limitations. Because this is a scoping review, we did not systematically assess the quality of individual studies. The studies reviewed may vary in quality or contain potential biases or methodological limitations. Therefore, this study is not intended to assess the quality of the evidence supporting informatics-based interventions on the given perinatal health conditions. Additionally, the nature of a scoping review is not to analytically aggregate studies so as to make claims about effect sizes. When additional, more comparable studies have accumulated, further research is needed to examine which informatics interventions are most effective and the size of the impact on maternal and perinatal health.

In addition, we only considered published studies that tested an informatics intervention and presented health-related outcomes. We excluded many studies because they tested feasibility or acceptability. It is possible that such excluded feasibility studies will eventually test health-related outcomes. Overall, this scoping review summarizes trends in the populations, geographic areas, technologies, and conditions targeted by informatics interventions tested and disseminated in the available literature.

### Conclusions

This scoping review paints a picture of the landscape of informatics interventions aimed at preventing and treating maternal morbidity. Most interventions identified in this study were tested in North America, East Asia and the Pacific, or Western Europe. Most tested either smartphone/tablet applications or telehealth/telemedicine, and most technologies tested for new mental health conditions and GDM. Almost all the studies tested technologies on populations of women and reported medical, mental health, and patient behavior outcomes. Results suggest that there may be opportunities to use informatics technologies to target providers who treat women as well as conditions such as postpartum hemorrhage that are more likely to lead to mortality. As the landscape of informatics applications in health care continues to expand, maternal health is poised to be an important target of these interventions.

## Appendix

Multimedia Appendix 1 PRISMA-ScR (Preferred Reporting Items for Systematic Reviews and Meta-Analyses Extension for Scoping Reviews) checklist.

## Abbreviations

AI: artificial intelligence
CDC: Centers for Disease Control and Prevention
GDM: gestational diabetes mellitus
HCAHPS: Hospital Consumer Assessment of Healthcare Providers and Systems
PCC: Population, Concept, and Context
PRESS: Peer Review of Electronic Search Strategies
PRISMA-ScR: Preferred Reporting Items for Systematic Reviews and Meta-Analyses Extension for Scoping Reviews
PTB: preterm birth
SMM: severe maternal morbidity

## Data Availability

The search strategies used in this study are openly available at searchRxiv [11,109-111].
